# NADH Fluorescence Lifetime Imaging Microscopy Reveals Selective Mitochondrial Dysfunction in Neurons Overexpressing Alzheimer’s Disease–Related Proteins

**DOI:** 10.3389/fmolb.2021.671274

**Published:** 2021-06-14

**Authors:** Moritz A. Niederschweiberer, Patrick M. Schaefer, Larry N. Singh, Ludwig Lausser, Devyani Bhosale, Raphael Hesse, Enrico Calzia, Hans A. Kestler, Angelika Rueck, Douglas C. Wallace, Bjoern von Einem, Christine A. F. von Arnim

**Affiliations:** ^1^ Department of Neurology, Ulm University, Ulm, Germany; ^2^ Department of Neurology and Experimental Neurology, Charité – Universitätsmedizin Berlin, Corporate Member of Freie Universität Berlin and Humboldt-Universität zu Berlin, Berlin, Germany; ^3^ Center for Mitochondrial and Epigenomic Medicine, Children’s Hospital of Philadelphia Research Institute, University of Pennsylvania, Philadelphia, PA, United States; ^4^ Institute of Medical Systems Biology, Ulm University, Ulm, Germany; ^5^ Department of Pharmaceutical Sciences, Philadelphia College of Pharmacy, University of the Sciences, Philadelphia, PA, United States; ^6^ University Medical School, Institute of Anesthesiological Pathophysiology and Process Engineering, Ulm, Germany; ^7^ Core Facility Confocal and Multiphoton Microscopy, Ulm University, Ulm, Germany; ^8^ Department of Pathology and Laboratory Medicine, University of Pennsylvania, Philadelphia, PA, United States; ^9^ Division of Geriatrics, University Medical Center Göttingen, Göttingen, Germany

**Keywords:** mitochondria, energy metabolism, Alzheimer’s disease, NADH, redox imaging, amyloid beta

## Abstract

Alzheimer’s disease (AD), the most prevalent form of dementia, affects globally more than 30 million people suffering from cognitive deficits and neuropsychiatric symptoms. Substantial evidence for the involvement of mitochondrial dysfunction in the development and/or progression of AD has been shown in addition to the pathological hallmarks amyloid beta (Aβ) and tau. Still, the selective vulnerability and associated selective mitochondrial dysfunction cannot even be resolved to date. We aimed at optically quantifying mitochondrial function on a single-cell level in primary hippocampal neuron models of AD, unraveling differential involvement of cell and mitochondrial populations in amyloid precursor protein (APP)-associated mitochondrial dysfunction. NADH lifetime imaging is a highly sensitive marker-free method with high spatial resolution. However, deciphering cellular bioenergetics of complex cells like primary neurons has still not succeeded yet. To achieve this, we combined highly sensitive NADH lifetime imaging with respiratory inhibitor treatment, allowing characterization of mitochondrial function down to even the subcellular level in primary neurons. Measuring NADH lifetime of the same neuron before and after respiratory treatment reveals the metabolic delta, which can be taken as a surrogate for cellular redox capacity. Correlating NADH lifetime delta with overexpression strength of Aβ-related proteins on the single-cell level, we could verify the important role of intracellular Aβ-mediated mitochondrial toxicity. Subcellularly, we could demonstrate a higher respiration in neuronal somata in general than dendrites, but a similar impairment of somatic and dendritic mitochondria in our AD models. This illustrates the power of NADH lifetime imaging in revealing mitochondrial function on a single and even subcellular level and its potential to shed light into bioenergetic alterations in neuropsychiatric diseases and beyond.

## Introduction

Neurons are highly complex and specific cells with synapses as their key structures, requiring a high energy supply to ensure neurointegrity and fast neurotransmission in the central nervous system. In the adult brain, approximately 90 percent of the energy supply in the form of ATP is provided by mitochondria (Harris et al., 2012).

Thus, mitochondrial dysfunction is found to be an early and crucial feature (Swerdlow et al., 2014) or even an underlying cause (Coskun et al., 2012) for neuropsychiatric and neurodegenerative diseases such as Alzheimer’s disease (AD) (Swerdlow and Khan, 2004; Grimm et al., 2016).

Aggregated forms of amyloid beta (Aβ) are pathological hallmarks of AD and were shown to be neurotoxic and affect mitochondrial function. Aβ derives from proteolytic processing of the amyloid precursor protein (APP) by beta-secretase (BACE1) and gamma-secretase. A mutation of the APP was found in families with early onset familial AD cases in Sweden (Mullan et al., 1992). This so-called APP Swedish mutation (APPswe) increases the APP cleavage by BACE1, which results in 6- to 8-fold level of Aβ compared to the normal wild-type APP (Citron et al., 1992).

In fact, Aβ was also found in the mitochondria (Caspersen et al., 2005) and is thought to exert its toxic effects locally (Schaefer et al., 2016), which could be an explanation for the selective vulnerability found in AD (Mattsson et al., 2016; Marzesco et al., 2016). Selective vulnerability on the cellular level was demonstrated by the selective loss of *α*-ketoglutarate dehydrogenase–enriched neurons in AD (Ko et al., 2001). Likewise, mitochondrial pools have been found to be affected differently, demonstrated by morphological changes in synaptic mitochondria (Balietti et al., 2013). However, a valid method to examine mitochondrial function in neurons on a cellular or subcellular level is still missing.

Nicotinamide adenine dinucleotide (NADH) fluorescence lifetime imaging microscopy (FLIM) has been shown to encode mitochondrial function, generally speaking with a longer NADH lifetime corresponding to higher respiration and a shorter NADH lifetime to lower respiration (Lakowicz et al., 1992).

However, the respiratory information can be confounded by numerous factors (Schaefer et al., 2018; Bartolomé and Abramov, 2015), such as nicotinamide adenine dinucleotide phosphate (NADPH) (Blacker et al., 2014), protein composition, or pH (Ogikubo et al., 2011), causing different absolute NADH lifetimes even in the absence of respiration.

In this study, we combine NADH FLIM and respiratory inhibitor treatment to introduce the metabolic delta, a robust parameter that allows differentiating mitochondrial function in primary hippocampal neurons on the single-cell level and between the somatic and neuritic mitochondrial pool. We apply NADH FLIM to primary hippocampal neurons overexpressing APP, APPswe, and BACE1 proteins, in order to reveal their effect on mitochondrial function down to the subcellular level. This enables uncovering crucial mechanisms of selective mitochondrial dysfunction in neuropsychiatric diseases such as Alzheimer’s disease.

## Methods

### Preparation of Primary Hippocampal Neurons

Primary hippocampal neurons (PHNs) were prepared from brains of C57BL/6J (JAX 000664) embryonic mice (E18), as described previously (Kaech and Banker, 2006; Hesse et al., 2018). Briefly, the pregnant mouse was sacrificed by cervical dislocation, and the embryos were extracted and decapitated. Hippocampi were dissected from the brains, the meninges were removed, and the cells were dissociated by trypsinization (0.25%) for 20 min at 37°C. The dissociated cells were resuspended in serum-free Neurobasal™ Medium (Thermo Fisher Scientific, Waltham, MA, United States) supplemented with 10% B27 Supplement (Thermo Fisher Scientific) and 0.5 mM L-glutamine, and passed through a 100-μm cell strainer (Corning, NY, United States). Cells were counted and seeded into poly-l-lysine–coated culture dishes at a density of 0.8 × 10⁴ cells cm^−2^ on 96-well plates, 4 × 10⁴ cells cm^−2^ on 24-well plates, and 6.6 × 10⁴ cells cm^−2^ on 6-well plates. After 45 min, the medium was replaced completely by a medium of the same kind to reduce astroglial growth. Cells were maintained at 37°C in the presence of 5% CO_2_ and 10% O_2_ in a humidified incubator. Partial media change was performed at day *in vitro* (DIV) 7 with discharge of 20% and recharge of 30% of the media. Transduction was performed together with partial media change at DIV7. Day of use in all experiments was DIV14.

### Expression Constructs

pUltra-hot is a lentiviral vector backbone for bi-cistronic expression of the gene of interest and the fluorescent reporter mCherry under the control of a human ubiquitin promoter. pUltra-hot was a gift from Malcolm Moore (plasmid #24130, Addgene, Watertown, MA, United States) and served as control plasmid and as backbone for the creation of APP pUltra-hot, APPswe pUltra-hot, BACE1 pUltra-hot, and Mt-SypHer pUltra-hot. BACE1 pUltra-hot was generated in our lab by subcloning BACE1 cDNA into the BamHI/XbaI cleavage sites of pUltra-hot. APP pUltra-hot was generated in our lab by subcloning APP695 cDNA into the XbaI/XmaI cleavage sites of pUltra-hot. APPswe pUltra-hot was also generated in our lab. We used the QuikChange Lightning Site-Directed Mutagenesis Kit (plasmid #210518-5, Agilent Technologies, Santa Clara, CA, United States) on APP pUltra-hot to insert a double-point mutation at the N-terminus of beta-amyloid (exon 16), called the Swedish mutation. SypHer mt (plasmid #48251, Addgene) was a gift from Nicholas Demaurex. SypHer mt allows the expression of a pH-sensitive ratiometric cpYFP derivative that contains two mitochondrial matrix localization sequences at its N-terminus. SypHer mt pUltra-hot was generated in our lab by subcloning SypHer mt into the EcoRI/NheI of pUltra-hot.

psPAX2 (plasmid #12260, Addgene) and pMD2.G (plasmid #12259, Addgene) were gifts from Didier Trono. psPAX2 is a packaging plasmid encoding for HIV-1 gag/pol sequences under the control of a SV40 promoter. pMD2.G is an envelope-expressing plasmid encoding for VSV-G glycoprotein under the control of a CMV promoter.

### Lentiviral Transduction System: Production, Testing, and Transfection

The lentiviral expression vector pUltra-hot is deleted for all genes associated with packaging or replication of the virus. Just the information for bacterial replication, the terminal recombination sequences, and the packaging signal is left. Thus, this third-generation vector represents a very safe lentiviral system as the virus particles are not able to replicate. For the virus production, Lenti-X™ 293 T cells (Takara Clontech, Mountain View, CA, United States) were seeded in high-glucose Dulbecco’s Modified Eagle’s Medium (DMEM) (Gibco) supplemented with 10% fetal calf serum (FCS) and 1% penicillin/streptomycin (P/S) and were co-transfected (calcium phosphate transfection method) with psPAX2, pMD2.G, and with the construct of interest (pUltra-hot, APP pUltra-hot, APPswe pUltra-hot, BACE1 pUltra-hot, or SypHer mt pUltra-hot). 6 h after transfection, the medium was changed to remove the transfection reagent. The cells were incubated at 37°C with 5% CO_2_ for 48 h. Success of transfection was controlled visually with a fluorescence microscope (Axiovert 2000; Carl Zeiss, Jena, Germany). Afterward, the transfection-conditioned medium was collected and filtered using a 0.2-μm sterile filter (Sarstedt, Nuembrecht, Germany). The conditioned medium was transferred to 38.5 ml Beckman Ultra-Clear™ tubes containing 3 ml 20% sucrose and spun for 2.5 h at 24,000 rpm (98,205 rcf) and 4°C (accelerate: 7/brake: 7) using a SW 32 Ti rotor and an Optima XPN-90 ultracentrifuge (both Beckman Coulter). The supernatant was discarded, and virus was resuspended in Dulbecco’s phosphate-buffered saline (Gibco™ Thermo Fisher Scientific) (DPBS), aliquoted, and stored at −20°C until use.

For testing of our lentiviral transduction system, the human embryonic kidney cell line HEK293 (DSMZ, ACC305, obtained 2008) was cultured in 75 cm^2^ flasks under standard cell culture conditions in high-glucose DMEM (supplemented with 10% FCS and 1% P/S). HEK293 cells were transduced with lentiviruses of pUltra-Hot, APP, APPswe, BACE1, and SypHer mt constructs in standardized serial dilution. After 5 days of cultivation, expression strength of pUltra-hot viruses was controlled by flow cytometry BD FACSCALIBUR™ (Becton Dickinson, Franklin Lakes, NJ, United States). Fluorescence of the reporter protein mCherry was detected as the mean FL4 channel intensity. For testing of the expression strength of SypHer mt, transduced HEK293 cells were resuspended in calibration solution with pH of 7.4 added with 25 μM of the K^+^/H^+^ ionophore nigericin followed by FACS analysis likewise.

### ATP Level Determination

PHNs were cultured on 96-well plates and transduced as described earlier with lentiviruses of APP, APPswe, and BACE1 with at least three technical replicates per independent experiment (n = 4). ATP levels were measured with ATPlite (Assay kit 6016943, PerkinElmer, Waltham, MA, United States), a highly sensitive ATP-monitoring luminescence assay kit following the instructions of the manual. We added antimycin A (AA, 5 µM) acutely to half of the samples to distinguish ATP deriving from glycolysis and respiration. Readout was performed with a PerkinElmer VICTOR X3 Multilabel Plate Reader following a luminometric top reading protocol at height of 13 mm with no filter.

### Mitochondrial Mass Determination

PHNs on 96-well plates were cultured and transduced as described earlier with at least five technical replicates per independent experiment (n = 5). Mitochondrial mass was measured with staining of 50 mM fluorescent MitoTracker Green FM dye (Thermo Fisher Scientific) diluted in neurobasal medium. Readout was performed using CLARIOstar (BMG Labtech, Ortenberg, Germany), a multichromatic fluorescence plate reader with excitation 480 nm and emission 530 nm. To avoid bleed-through, a dichroic filter at 504 nm was used.

### Mitochondrial Membrane Potential

PHNs on 96-well plates were cultured and transduced as described earlier with at least six technical replicates per independent experiment (n = 8). For the determination of mitochondrial membrane potential, JC-1 dye (T3168, Thermo Fisher Scientific) was used. The staining solution was prepared by mixing JC-1 stock (5 mg ml^−1^) with dimethyl sulfoxide (DMSO) in a ratio of 1:3 and subsequent dilution in further mentioned DMEM (supplemented with 10% FCS and 1% P/S) to a final concentration of 2.5 μg ml^−1^; cells were stained for 15 min at 37°C in the dark.

We uncoupled half of the samples using the ionophore carbonyl cyanide 4-(trifluoromethoxy) phenylhydrazone (FCCP) (7.5 µM) at the same time of staining. Readout was performed using BMG CLARIOstar, a multichromatic fluorescence plate reader. We used 488 nm excitation and emission of both 540 and 600 nm for ratiometric determination of mitochondrial membrane potential. To avoid bleed-through, we used two dichroic filters at 513 and 543 nm, respectively.

### High-Resolution Respirometry

PHNs on 6-well plates were cultured and transduced as described earlier with at least two technical replicates per independent experiment (n = 6). High-resolution respirometry was performed in an Oxygraph-2k system (Oroboros Instruments, Innsbruck, Austria) calibrated to air (gain for oxygen sensor was set to 4) with MIRO-5 (Oroboros Instruments), a mitochondrial respiration medium. Cells were added to the two stirred (750 rpm) chambers, which were sealed to obtain a closed system. Decreasing oxygen concentration in the chambers resembled cellular oxygen consumption. Substrate-uncoupler-inhibitor titration (SUIT) protocol for permeabilized cells was used. For permeabilization of the cells, PHNs were scratched down in 2.5 ml MIRO-5, which resulted in a good permeabilization indicated by the absence of routine respiration and reaction to hydrophilic substrates. First, the complex I substrates pyruvate (5 mM), malate (2 mM), and glutamate (10 mM) were added followed by adenosine diphosphate (ADP, 2.5 mM) to allow coupled respiration through complexes I, III, IV, and V (labeled as C I respiration). Subsequently, cytochrome c (10 μM) was added to check for the integrity of the inner mitochondrial membrane (state not displayed). Addition of succinate (50 mM) and octanoylcarnitine (0.5 mM) provided the maximum capacity of the oxidative phosphorylation (OxPhos capacity). Titration of FCCP with a final concentration of ∼3 μM uncoupled the mitochondria, allowing evaluation of the capacities of complexes I–IV without potential limitation by complex V, which is called the electron transport system capacity (ETS capacity). Afterward, 1 μM rotenone was added to block complex I and measure complex II through IV activity (C II).

Finally, the addition of 5 μM AA blocked mitochondrial respiration completely, showing residual non-mitochondrial oxygen consumption (ROX), which all respiratory states were corrected for.

All states were normalized to citrate synthase activity (CSA). Analysis of the measurements was performed using DatLab version 5.1.0.20 (Oroboros Instruments). Time intervals were drawn at the stable plateaus of oxygen flux quantifying the mean oxygen consumption of the respiratory states, which were corrected for ROX afterward. All uncouplers and inhibitors were bought from Sigma-Aldrich. After the experiment, the samples were directly frozen using liquid nitrogen and stored at −20°C.

### Citrate Synthase Activity

The determination of citrate synthase activity was performed in selfsame previously frozen samples of high-resolution respirometry using the CSA assay kit (MAK193, Sigma Aldrich, St. Louis, MO, United States), which is based on a colorimetric enzyme-coupled reaction (412 nm). Therefore, former mentioned samples of permeabilized PHNs were slowly thawed, split into three, and transposed to a 96-well plate. Readout was performed using Mithras LB 940 (Berthold Technologies, Bad Wildbad, Germany), a multichromatic fluorescence plate reader. After 10 min of background analysis, substrate was added, and enzyme activity was measured for 30 min.

### NADH FLIM

PHNs seeded on 24-well plates were cultured and transduced with pUltra-hot lentiviruses and in addition SypHer mt as described earlier. Imaging was performed at 37°C, at atmospheric CO_2_, and in Tyrodes buffer (pH 7.4, HEPES-buffered), which was applied 20 min prior to the experiment.

Lifetime imaging of NADH autofluorescence was performed on a laser scanning microscope (LSM 710, Carl Zeiss) equipped with a pulsed (80 MHz, 100 fs pulse width) titanium–sapphire laser (Mai Tai AX HPDS, Spectra Physics). Endogenous NADH fluorescence was induced *via* two photon excitations at 730 nm with a maximal power of 5 mW at the output of the objective lens and detected using a 460/60 emission filter (AHF Analysentechnik, Tübingen, Germany). Time-correlated single photon counting (TCSPC) was performed by the hybrid detector HPM-100–40 (Becker and Hickl GmbH, Berlin, Germany) coupled to the NDD port of the LSM 710. For each photon, the location within the scanning area and the time of the photon within the laser pulse period are determined using an image size of 512 × 512 pixel and a temporal resolution of 256 time channels within a pulse period of 12.5 ns Collection time was set to 60 s, with a pixel dwell time of ≈15 µs. An area of 132.5 × 132.5 μm^2^ was scanned using an EC Plan-Neofluar 40 × /1.30 oil objective (Carl Zeiss).

TCSPC data were recorded using SPCM 9.6 (Becker and Hickl GmbH) and analyzed using SPCImage 5.0 (Becker and Hickl GmbH). The instrument response function was determined automatically by the software according to bH TCSPC handbook (Becker, 2015). A bi-exponential decay with fixed lifetime components of 400 and 2,500 ps for free and protein-bound NADH, respectively, was assumed to reduce the amount of photons needed for a good calculation of the mean lifetime (τmean). Both the lifetimes for free and protein-bound NADH were previously evaluated on a smaller set of cells (data not shown, n = 3, > 50 neurons) and did not change significantly upon AA (5 µM) treatment, indicating the lifetime components to be relatively stable. Thus, using τmean with fixed lifetime components provides a comparable readout to the more common ratio of free to bound NAD(P)H (a1/a2) but allows an improved fitting at low photon numbers.

τmean of a pixel was calculated using a binning factor of 2. Fitting of the calculated lifetime curve was checked by evaluating the mean χ^2^, which was below 1.2. Fitting-free analysis using the phasor approach was evaluated but proved difficult to be combined quantitatively with the metabolic delta and the pH correction.

Subcellular analysis of NADH lifetime was performed by calculating τmean of regions of interests (ROIs), drawn manually, in an image. At least one somatic region and one region of dendrites were labeled per picture. The ROIs were never overlapping. Mitochondrial pools could not be analyzed separately due to a poor spatial separation of mitochondria/cytoplasm in the soma of neurons.

For the determination of the metabolic delta, PHNs were first measured in untreated conditions, whereat the exact coordinates of the microscope’s position system on the three axes (x/y/z) were noted. Then, AA at a final concentration of 5 μM was added to block mitochondrial respiration completely, and the exact positions of the prior images were focused and imaged again within 30 min. The difference between the τmean in untreated condition and after AA accounts for the metabolic delta.

### Determination of Matrix pH

For imaging of mitochondrial matrix pH, we used a lentiviral vector containing SypHer mt targeted to the mitochondria as described previously (Schaefer et al., 2017).

SypHer mt was excited at two different excitation wavelengths (405 and 488 nm), and its emission was recorded at 525 nm. For calibration, PHNs on 24-well plates transduced with SypHer mt as described earlier were measured in calibration buffer supplemented with 25 µM of the K^+^/H^+^ ionophore nigericin at pH 7.0 and 8.0. Ten images of each pH condition were recorded for each independent experiment (n = 5), and the emission intensity ratio for both excitation wavelengths were used for calibration prior to each NADH pH imaging experiment, respectively.

### Determination of Expression Strength

For the determination of expression strength of APP, BACE1, and APPswe, imaging of the red fluorescent protein mCherry was used at 561 nm excitation and 645 nm emission. Imaging was performed immediately prior to matrix pH imaging in a different channel.

### Combined NADH FLIM/pH Imaging

For combined imaging of NADH autofluorescence and mitochondrial pH, transduced cells were seeded and cultivated according to the FLIM protocol. For imaging of NADH autofluorescence, a 460∕60 emission filter and a 436∕20 emission filter were both used to exclude SypHer mt to be detected in the FLIM channel. NADH FLIM was imaged first, followed by SypHer mt; both were recorded as described above.

For pH correction of τmean, we calculated the difference of mitochondrial matrix pH of every neuron to the arithmetic mean mitochondrial matrix pH of all untreated neurons. Multiplying this delta mitochondrial matrix pH with the effect strength of pH on τmean (113.6 ps/pH unit) revealed the “pH-induced lifetime alteration” for every neuron. This was subtracted from the measured τmean to reveal the pH-corrected mean NADH lifetime. The pH-corrected metabolic delta was calculated as the difference between the pH-corrected τmean in untreated vs. AA-treated neurons.

### Statistical Analysis

Statistical analysis was performed using GraphPad Prism 5 (GraphPad Software, San Diego, CA, United States). D’Agostino and Pearson omnibus normality test was used to check for a Gaussian distribution of the data (significance level *α* < 0.05). For data in which all groups passed the normality test, an unpaired two-tailed *t* test (for two groups) or a one-way analysis of variance (ANOVA) using pairwise multiple comparisons with the Bonferroni correction (for more than two groups) was performed to check for significance. For data in which at least one group did not pass the normality test, a Kruskal–Wallis test with Dunn’s multiple comparison tests was performed. Paired values were analyzed using the Friedman test and Dunn’s multiple comparison test. Significance between all pairs was evaluated if not described otherwise, but only significant differences were indicated graphically (*p < 0.05, **p < 0.01, ***p < 0.001).

To make full use of the single-cell resolution of NADH FLIM and allow correlations with transduction strength, we used individual soma as input into the statistics. As dendrites within an image section could not be assigned to individual soma beyond doubt, we used individual image sections as input for the statistics when comparing soma and dendrites. To account for day-to-day variability and correct for batch effect, all data using individual neuron/image input were normalized. The measures within each day were Z-transformed to mean 0 and standard deviation 1, for each of soma vs. dendrite and each of the expressed proteins. These Z-transformed values were then inverse-transformed by multiplying by the “global standard deviation” and adding the “global mean.” The “global mean” and “global standard deviation” are the mean and standard deviation of the measures for each of soma vs. dendrite and each of the expressed proteins combined for all days, respectively.

For comparisons between cell compartments as well as between overexpressed proteins, two-way mixed ANOVA with repeated measures was used, and implemented in the R packages afex and emmeans. In case of significance (*p* < 0.05) within soma and dendrites, *post hoc* tests were applied using pairwise Mann–Whitney tests and the Bonferroni multiple testing correction. Paired Mann–Whitney *post hoc* tests with Bonferroni multiple testing corrections were performed for overexpressed proteins between soma and dendrites.

Statistical analyses for correlations, including Kendall-Tau and Pearson R, were computed using R v3.5.2^1^, and plots were generated using ggplot2^2^. The “self *p*-value” plots were computed by first sorting all points by x-coordinate. A reference set of points comprising the points with the smallest 21 x coordinates was then generated. A window of the next 21 points in order of x coordinate was then compared to the reference window of points using a permutation Wilcoxon–Mann–Whitney test using the R zoo package. The window was then slid along the *x*-axis by adding the point with the next greatest x coordinate and removing the point with the smallest x coordinate. The *p*-value was computed using a permutation test version of the Wilcoxon–Mann–Whitney test in the R coin package. For visualization of the nonlinear correlations, a smooth curve was fitted through the points in the scatterplot, using local regression (loess). Loess curves were computed using the R loess function with default options and default span of 0.75.

## Results and Discussion

### Respiratory Inhibitory Treatment Reveals Metabolic Delta

**FIGURE 1:**
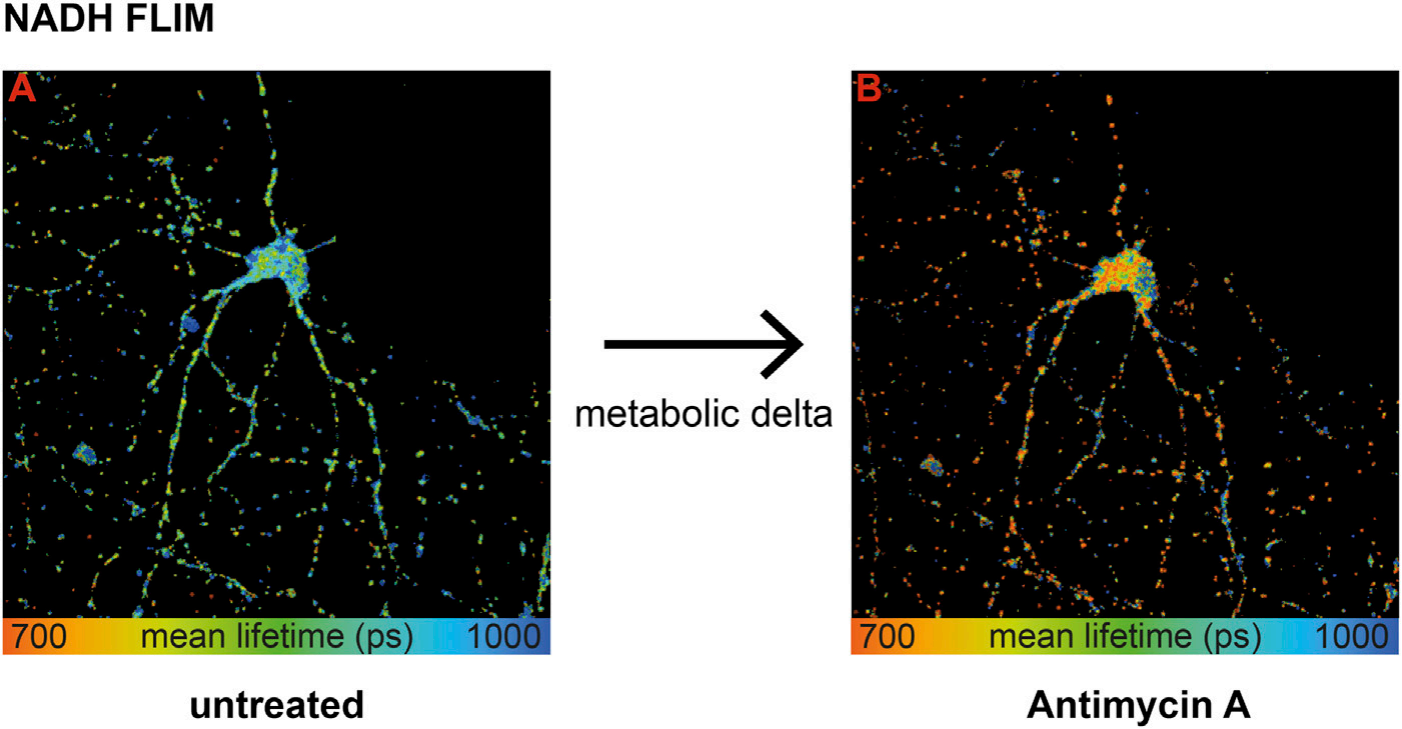
Wild-type primary hippocampal neurons (PHNs) were imaged and analyzed for NADH lifetime before **(A)** and after **(B)** antimycin A treatment. Images A and B display the mean NADH lifetime (Ƭmean) of neurons using NADH FLIM. The change in Ƭmean between the two conditions untreated and AA is called metabolic delta. Ƭmean is false-color coded with the corresponding color palette shown below the images. Metabolic delta for the pH effects provides a surrogate for mitochondrial respiration.

To extract mitochondrial respiration from the NADH lifetime, the most important part is to eliminate variations in the baseline due to causes other than respiration (Bartolomé and Abramov, 2015). Measuring the same neuron before (Figure 1, image A) and after treatment (Figure 1, image B) with an inhibitor of the respiratory chain–like antimycin A (AA) allows determining the change in the NADH lifetime, further called metabolic delta. It is mostly dependent on the change in mitochondrial respiration, while all parameters that are not affected by an acute inhibitor treatment are corrected for (Schaefer et al., 2018). Thus, a large metabolic delta indicates high respiration.

To sum up, influencing factors like protein composition, NADPH, or viscosity are eliminated with the metabolic delta, which can be taken as a surrogate marker for mitochondrial respiration. Thus, NADH FLIM, before and after respiratory inhibitor treatment on the same neuron, allows extracting mitochondrial respiration with high spatial resolution.

### Overexpression of APPswe in Neurons Results in a Mild Mitochondrial Defect

To address the question of selective mitochondrial dysfunction in AD-affected neurons, we overexpressed human APP, APPswe, and BACE1, all of which have been demonstrated to cause mitochondrial dysfunction in different model systems (Rhein et al., 2009; Walls et al., 2012; Findlay et al,. 2015) in primary hippocampal neurons. Using a bi-cistronic vector that expresses the red fluorescent protein mCherry and the protein of interest to a similar absolute amount (Supplementary Figure S1A) provides a fluorescent readout of the overexpression strength in single neurons. We targeted for similar mean overexpression strength between the proteins at a level that showed about a 2-fold increase in Aβ levels in APP neurons (Supplementary Figures S3A–C). We further controlled that viral transduction alone does not induce respiratory effects (Supplementary Figure S1C).

**FIGURE 2:**
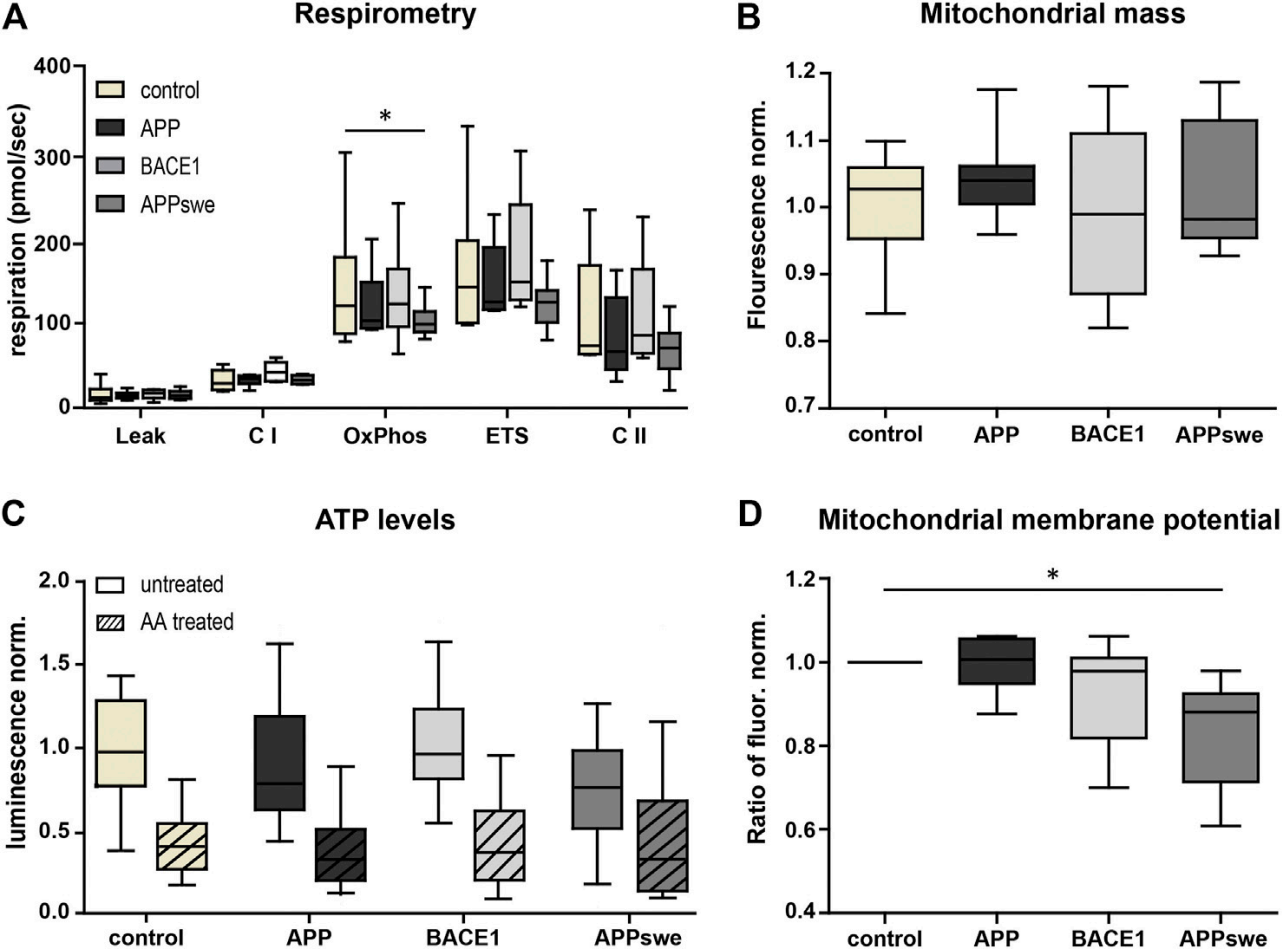
Metabolic characterization shows decreased OxPhos capacity and mitochondrial membrane potential in APPswe-transduced primary hippocampal neurons. **(A)** High-resolution respirometry of permeabilized neurons was performed in an Oroboros Oxygraph-2k. All respiratory states were normalized to citrate synthase activity. Significance vs. control was calculated using the Friedman test and Dunn’s multiple comparison test (n = 6, each in duplicates). **(B)** Mitochondrial mass measured as Mitotracker Green fluorescence intensity normalized to control. Statistics were calculated using ANOVA with Bonferroni’s multiple comparison test (n = 5, each in quintuplicates). **(C)** ATP levels were determined using luminescence assay kit ATPlight. AA (5 mM) was added acutely to distinguish ATP deriving from glycolysis and respiration. Fold change to pUltra-hot control was calculated. Significance vs. control was calculated using the Kruskal–Wallis test with Dunn’s multiple comparison test (n = 4, each in at least triplicates). **(D)** Mitochondrial membrane potential was measured as fold change of the red/green fluorescence intensity ratio of untreated over FCCP-treated neurons (7.5 µM) using the potentiometric dye JC-1 (n = 8, each in at least sextuplicates). Significance vs. control was calculated using the Friedman test and Dunn’s multiple comparison test. All box plots show interquartile range, with the line indicating the median and the whiskers 10–90%.

First, we characterized mitochondrial capacity biochemically using standardized methods. Respirometry showed a consistent tendency for a reduced OxPhos capacity, ETS capacity, and complex II (CII) respiration in APP and APPswe neurons (Figure 2A), with APPswe reaching significance in OxPhos capacity compared to control.

In contrast, BACE1 overexpressing neurons did not show a reduced respiration nor does complex I (CI)-respiration in any of the conditions. Next, we measured mitochondrial mass using the well-established Mitotracker Green FM. This did not reveal any significant differences (*p* = 0.1575) (Figure 2B), indicating that the reduced respiration is not due to a reduced mitochondrial mass. Also, consistent with the respirometry results, the total ATP levels were lower in APPswe neurons (Figure 2C), although this effect did not reach significance (*p* = 0.09). However, upon acute AA pretreatment, the ATP levels converged (Figure 2C), indicating that mitochondrial ATP production is reduced in APPswe neurons (Supplementary Figure S1D). Likewise, mitochondrial membrane potential decreased significantly (*p* = 0.03) in APPswe neurons (Figure 2D).

To sum up, APPswe and to a smaller extent also APP overexpression in neurons resulted in a mildly reduced mitochondrial respiration and ATP production, while no effects could be detected upon BACE1 overexpression. In general, the mitochondrial function was only moderately affected by the overexpression strength chosen, allowing testing NADH-matrix pH imaging on early pathophysiological conditions of the disease with mostly preserved overall mitochondrial capacity.

### Mitochondrial Matrix pH Alterations Partially Mask the Effect of a Lower Mitochondrial Respiration in NADH FLIM

**FIGURE 3:**
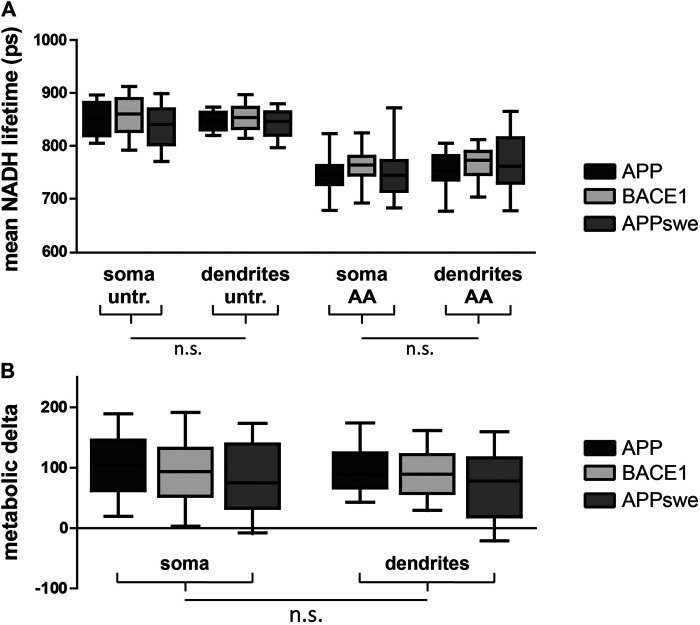
PHNs show similar NADH lifetime and metabolic delta in soma and dendrites. Mean NADH lifetime **(A)** and metabolic delta **(B)** (surrogate for mitochondrial respiration) in soma and dendrites of primary hippocampal neurons overexpressing APP (black), BACE1 (light gray), or APPswe (dark gray). Calculated as the difference between untr and AA of **(A)**. Significances were analyzed between conditions (APP, BACE1, and APPswe) and between regions (soma and dendrites) of the same condition and combined conditions (APP, BACE1, and APPswe pooled together) on the single-image level using two-way mixed ANOVA and the Mann–Whitney *post hoc* test with the Bonferroni correction for multiple testing (n > 50 images). No significance was found for any aforementioned conditions. All box plots show interquartile range, with the line indicating the median and the whiskers 10–90%. **(C)** The effect size of matrix pH on τmean was quantified by correlating τmean with mitochondrial matrix pH in individual neurons pretreated with antimycin A (5 µM) (n = 5, >100 neurons, τ: −0.190, *p* = 0.002).

Next, we aimed at comparing NADH FLIM to the biochemical metabolic characterization in primary neurons overexpressing APP, BACE1, and APPswe. The mean NADH fluorescent lifetimes show the expected decrease upon AA treatment but are not significantly different between the overexpressed proteins or between soma and dendrites (Figure 3A). Calculating the metabolic delta as the difference between untreated and AA-treated neurons reveals a trend toward a lower metabolic delta in APPswe (Figure 3B), resembling the trends seen for the respirometry measurements (Figure 2A). One explanation for the minor effect size in bulk NADH FLIM compared to respirometry could be alterations in mitochondrial matrix pH (Schaefer et al., 2018).

To assess this possibility, we first assessed the global effect size of mitochondrial matrix pH alterations upon NADH lifetime by correlating both parameters in neurons with blocked mitochondrial respiration. We could demonstrate a significant negative correlation (Supplementary Figure S2B, *τ* = −0.190, *p* = 0.002), which is in accordance with the known pH effect on NADH lifetime (Schaefer et al., 2017).

As expected, we found a significant mitochondrial matrix acidification upon AA treatment (Supplementary Figure S2A). Interestingly, dendritic mitochondria showed a higher matrix pH both in untreated and AA-treated neurons, possibly indicating differences in the inner mitochondrial membrane leak between both mitochondrial pools.

We recently established that NADH lifetime can be corrected for the effects of a changing matrix pH to reveal an even more precise readout of mitochondrial respiration (Schaefer et al., 2018). Performing this correction reveals the highest metabolic delta in BACE1 and a significantly lower metabolic delta in APPswe, verifying our biochemical data of a lower mitochondrial respiration upon overexpression of APPswe (Supplementary Figures S2C,D). Furthermore, the lower metabolic delta in dendrites after pH correction suggests a lower mitochondrial respiration in the dendrites than the soma, which might be associated to a low endogenous activity of the neurons.

Taken together, mitochondrial matrix alterations partially mask the effect of a lower mitochondrial respiration in APPswe in bulk NADH FLIM.

### Somatic Mitochondria Demonstrate Significant Affection by Overexpression of AD-Related Proteins

While pH correction works nicely on a population of cells, it is much more challenging on the single-cell level due to a high variability of the pH readout and effect size of pH in each individual neuron (Supplementary Figure S2B). Thus, we wanted to know if the metabolic delta alone is sensitive enough to reveal mitochondrial differences on the single-cell level.

Using the fluorescence of the reporter protein mCherry as a surrogate for the overexpression strength allowed us to correlate mitochondrial function with overexpression strength of APP, BACE1, and APPswe. First, we verified that the 21 lowest overexpressing neurons of each condition have a comparable NADH lifetime and metabolic delta (Supplementary Figures S2E,F) between each other and compared to wild-type neurons, excluding mitochondrial toxicity due to secreted Aβ or SypHer mt.

**FIGURE 4:**
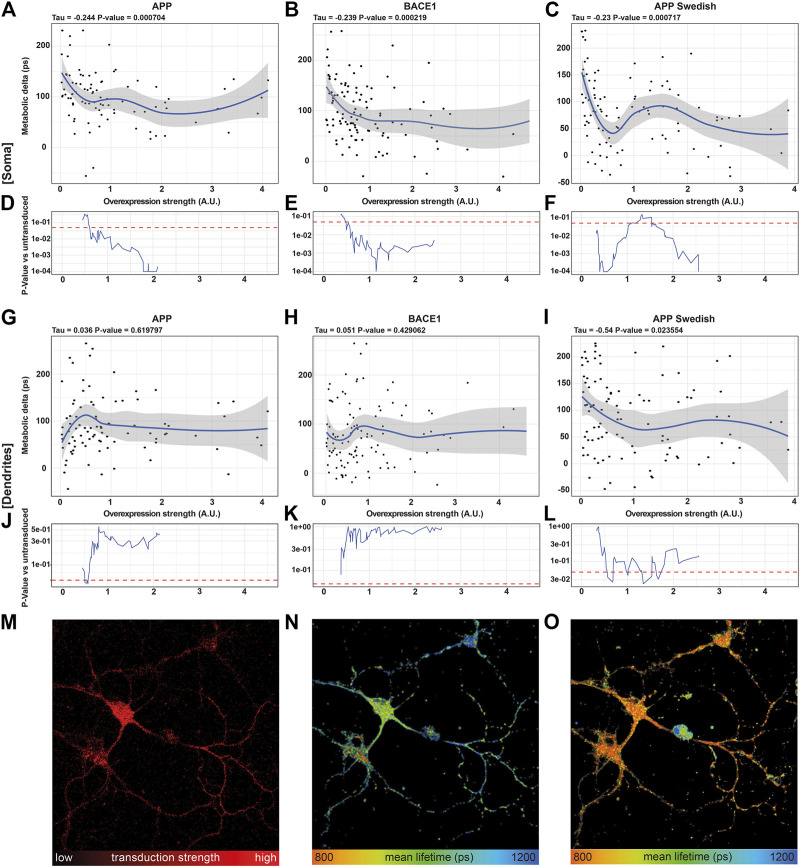
NADH FLIM Metabolic Delta shows dose-mitochondrial respiration correlates with APPswe overexpression strength in a dose-dependent manner. **(A–C, G–I)** Correlations of the metabolic delta in somatic **(A–C)** and dendritic regions **(G–I)** with the overexpression strength of APP (soma: τ = −0.244, *p* = 0.0007, dendrites τ = 0.036, *p* = 0.619,797), BACE1 (soma: τ = −0.239, *p* = 0.0002, dendrites: τ = −0.051, *p* = 0.429,062), and APPswe (soma: τ = −0.231, *p* = 0.0007, dendrites: τ = −0.54, *p* = 0.023,554), quantified as mCherry fluorescence intensity of APP **(A)**, BACE1 **(B)**, and APPswe **(C)** neurons on the single-cell level (n = 5, > 120 neurons). The blue line represents a local regression (loess) curve to visualize the nonlinear regression. **(D–F, J–l)** Significance indicated as *p*-value [p] vs. the 21 least transduced neurons was evaluated using a sliding window and a permutation test and plotted in **(D–F, J–O).** A representative image section of BACE1 neurons underlining the decrease in the metabolic delta with increasing overexpression strength. **(M)** Overexpression strength is displayed as the mCherry fluorescence intensity. NADH FLIM before **(N)** and after AA treatment **(O)** are false-color coded with the corresponding color palette shown below.

Correlating the metabolic delta in somata of single neurons with the overexpression strength of APP (*τ* = −0.244, *p* = 0.0007), BACE1 (*τ* = −0.239, *p* = 0.0002), and APPswe (*τ* = −0.231, *p* = 0.0007) revealed highly significant negative correlations for all overexpressed proteins, with smaller metabolic deltas upon a higher overexpression strength (Figures 4A–C). This can even be visualized in a representative image section, where the neuron with the highest BACE1 overexpression displays the shortest NADH lifetime and *vice versa* (Figure 4M,N). However, upon AA treatment, all somata show a comparably short NADH lifetime (Figure 4O). In contrast, no significant correlations of the NADH lifetime to overexpression strength (untreated: *p* = 0.64, AA: *p* = 0.26) were seen in pUltra-hot neurons with the tendency rather pointing toward an increased metabolic delta in highly transduced pUltra-hot neurons (Supplementary Figure S3C).

Interestingly, in dendrites, the correlation between metabolic delta and overexpression of Alzheimer proteins is reduced in APPswe (*τ* = −0.54, *p* = 0.02355) or even abolished in APP (*τ* = 0.036, *p* = 0.6198) and BACE1 (*τ* = 0.051, *p* = 0.42906) (Figures 4G–I). This indicates that mitochondrial respiration is predominantly affected in the soma as opposed to the dendrites and thereby underlines the need of subcellular differentiation of mitochondrial function in neurons.

### Rolling Window Analysis Revealing Triphasic Dose–Response in APPswe Overexpressing Neurons

Going into detail on the dose–response, we compared the 21 lowest overexpressing neurons to a sliding window moving along the overexpression strength. The resulting *p*-value curves reveal that already a 0.6-fold average overexpression of APP or BACE1 (Figures 4D,E) or 0.4 fold average overexpression of APPswe (Figure 4F) results in a significantly reduced respiration in the somatic region. This is surprising as already a weak overexpression reduces mitochondrial respiration, as opposed to a threshold model that would be expected for a direct inhibition of one of the complexes due to their reserve capacity. While the mitochondrial capacity was not reduced strongly (Figure 2A), it appears that the respiration under endogenous substrates is more affected, indicating an upstream downregulation of respiration by APP, BACE1, and APPswe (Hernandez-Zimbron et al. (2012); Zhao and Zhao (2013)), possibly *via* an increase of oxidative stress or inhibition of the TCA cycle (Kokoszka et al., 2001; Butterfield et al., 2013).

Interestingly, in somata of APPswe overexpressing neurons, this decrease in respiration did not continue exponentially with overexpression strength. Indeed, while APPswe neurons at a low average overexpression of 0.5 showed strongly reduced respiration (*p* = 0.0001), neurons with a mild average overexpression around 1.3 showed no significantly reduced respiration anymore (*p* = 0.1). It is unlikely that this is due to different neuronal subpopulations as we did not observe this pattern in BACE1 or pUltra neurons. A possible explanation could be changes in the intracellular transport with overexpression strength as already observed for BACE1 overexpression (Lee et al., 2005). This could result in differences in Aβ production, the Aβ species, in its secretion vs. intracellular accumulation (Schaefer et al., 2016; Devi and Anandatheerthavarada, 2010), or in its intracellular localization.

The rolling window analysis of the metabolic delta in dendritic regions showed no significant dose–response to BACE1 or APP overexpression. APPswe however showed a significantly reduced metabolic delta between 0.7 and 1.7 average overexpression strength, the same range in which the somatic effect is less pronounced. This effect could be due to subcellular routing of proteins in neurons affected by AD and further emphasizes local changes of mitochondrial function with overexpression strength. Finally, our findings underline the strength of NADH FLIM, deciphering cellular respiration on the subcellular level.

### NADH Metabolic Delta Deciphers Cellular Respiration with Subcellular Resolution in AD and Other Neuropsychiatric Diseases

NADH FLIM has been applied previously in neurons from transgenic animals in the context of AD (Dong et al., 2019; Theurey et al., 2019). Interestingly, a decrease of free/bound NADH was observed in these studies, which goes along with a general decrease in the NAD/NADH pool with aging. In contrast, we used a primary neuron culture model exhibiting core (but not all) hallmarks of AD and investigated the acute (within one week) effect of a slight overexpression of APP, BACE1, or APPswe on mitochondrial function. As it would be expected from an acute inhibitory effect on the mitochondrial respiration (Chakraborty et al., 2016), we observed an increase in free/bound NADH with increasing expression strength.

Combining NADH and acute respiratory inhibitor treatment allows the determination of the metabolic delta, which can be taken as a surrogate for cellular respiration. Taken together, this method enables deep insights into cellular bioenergetics with subcellular resolution even in complex cells as primary hippocampal neurons. Furthermore, this leaves the orange/red spectral range for use of additional dyes, for example, to correlate mitochondrial function with overexpression strength or similar parameters of interest. This is especially useful if only a subset of cells harbors mitochondrial alterations. In the present study, neither respirometry (Figure 2A) nor bulk NADH FLIM (Figure 3) was able to detect a reduction in mitochondrial function upon the mild overexpression of APP or BACE1. However, using the single-cell resolution of NADH FLIM and correlating it with the overexpression strength revealed mitochondrial toxicity of APP, BACE1, and APPswe, although for BACE1 and APP, only at a higher expression level. This emphasizes the broad application range of NADH FLIM for studying mitochondrial function on the single-cell level in multiple diseases.

With respect to Alzheimer’s disease, NADH FLIM showed a downregulation of mitochondrial respiration in primary hippocampal neurons already upon slight overexpression of APP, BACE1, or APPswe, most likely due to intracellular Aβ. Consequently, our study provides evidence on how even a physiologically possible increase in the expression of AD-related proteins can markedly alter mitochondrial metabolism and the mitochondrial pool affected. Of course, a cellular model such as the one used here cannot fully reflect the complex pathophysiological conditions we find in AD patients (e.g., aging, network dysfunction, blood brain barrier dysfunction, and inflammation). However, it has already been shown that primary hippocampal neurons serve as a valid model in AD research because they can be isolated directly from the tissue and retain *in vivo* properties. They are physiologically relevant and reflect the cell type affected in AD. Therefore, our results emphasize the potential of NADH FLIM, paving the way for reliable measurements in different neuronal cell types, for example, cortical neurons, which will allow to decipher mitochondrial dysfunction associated with Alzheimer’s disease and other neuropsychiatric disorders.

## Data Availability

The raw data supporting the conclusions of this article will be made available by the authors, without undue reservation.

## Supplementary Material

SUPPLEMENTARY FIGURE 1 | Overexpression of AD-related proteins using the lentiviral transduction system. **(A)** Overexpression of APP, APPswe, and BACE1 in HEK293 cells transduced with the pUltra-hot lentiviral system and detected by Western blotting. Rotiblock was performed for one hour at 4°C. mCherry, APP, and BACE1 overexpression was detected using a-tRFP, a-APP-c-term, and a-BACE1 antibody (all Sigma), respectively. β-actin as a loading control was detected using AC-15 α-β-actin antibody (Sigma). Anti-mouse/rabbit horseradish protein in PBS was used as second antibody. Developed with LAS 400 and Luminata Forte. **(B)** Quantification of intracellular Aβ and Aβ secretion rate in primary neurons transduced with the pUltra-hot lentiviral system using ELISA. Results were normalized to the mean values of control cells, which were set to 1. The mean values of 1 independent experiment measured in three technical replicates is shown. **(C)** High-resolution respirometry of permeabilized neurons was performed in an Oroboros Oxygraph-2k. The means of 6 (pUltra-hot) or 3 (wt) independent experiments are shown. Error bars indicate standard error of the mean. Significance versus control was calculated using t test. **(D)** Mitochondrially derived ATP levels were calculated as the difference between untreated and AA-treated neurons in Figure 2C normalized to pUltra-hot. Significance to control was calculated using the Kruskal–Wallis test with Dunn’s multiple comparison test (n = 4, each in at least triplicates).

SUPPLEMENTARY FIGURE 2 | pH-corrected metabolic delta mirrors respirometry results upon overexpression of AD-related proteins in PHNs. **(A)** Quantification of the mitochondrial matrix pH using mito-SypHer performed in combination with NADH FLIM. **(B)** Effect size of Matrix pH on NADH lifetime quantified as correlation between both parameters in antimycin A–treated neurons. **(C)** pH-corrected NADH lifetime before and after antimycin A (AA) treatment in soma and dendrites of primary hippocampal neurons overexpressing APP (black), BACE1 (light gray), or APPswe (dark gray). Calculated from the combined quantification of the mean NADH lifetime (Figure 3A) and mitochondrial matrix pH (Supplementary Figure S2A) with the pH effect size as determined in Supplementary Figure S2B (n = 5, >120 neurons). **(D)** pH-corrected metabolic delta calculated as the difference in the pH-corrected NADH lifetimes between untr and antimycin A–treated neurons (Supplementary Figure S2C). Significances were analyzed between conditions (APP, BACE1, APPswe, dashed lines) and between regions (soma and dendrites) of the same condition (dotted lines) and combined conditions (APP, BACE1, APPswe pooled together, solid lines) using the Kruskal–Wallis test with Dunn’s multiple comparison test. e/f) Mean NADH lifetime (e) and metabolic delta (f) of wild-type (WT) neurons compared to the 21 weakest overexpressing neurons for APP, BACE1, and APPswe (internal controls). Significances were analyzed between all conditions using the Kruskal–Wallis test with Dunn’s multiple comparison test. All box plots show interquartile range, with the line indicating the median and the whiskers 10–90%.

SUPPLEMENTARY FIGURE 3 | Expression of control vector does not affect the NADH lifetime. **(A,B)** Correlation of the mitochondrial matrix pH with the normalized overexpression strength of APP (black), BACE1 (light gray), and APPswe (dark gray), quantified as mCherry fluorescence intensity, on the single-soma level. The same neurons were imaged before **(A)** and after **(B)** antimycin A treatment (n=5, >120 somata). **(C)** Correlation of the mean NADH lifetime with expression strength of pUltra-hot control in untreated (black) and acute antimycin A–treated (gray) neurons on the single-soma level (n = 2, >20 somata). The lines in the scatterplots represent local regression (loess) curves to visualize the nonlinear regressions. Significance indicated as *p*-value [*p*] versus the 21 **(A,B)** or 9 **(C)** least transduced neurons was evaluated using a sliding window and a permutation test and plotted below the scatterplots.
