# Supplementary figures and images for: NADH Fluorescence Lifetime Imaging Microscopy Reveals Selective Mitochondrial Dysfunction in Neurons Overexpressing Alzheimer’s Disease–Related Proteins
Source: Front Mol Biosci. 2021 Jun 14;8:671274. doi: 10.3389/fmolb.2021.671274 (PMC8236706; doi:10.3389/fmolb.2021.671274)

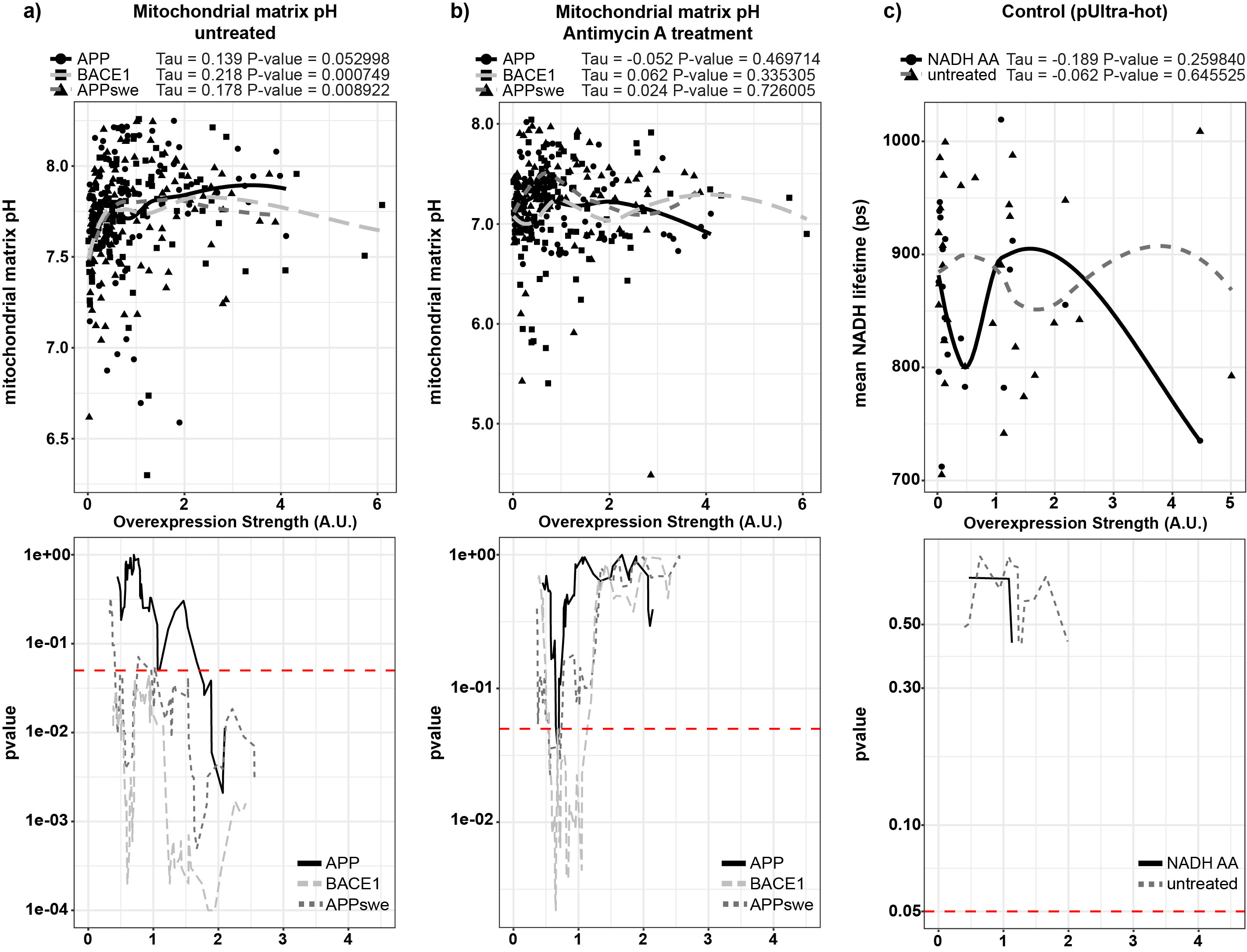

Supplement: Supplementary file 1 [file Image3.JPEG]

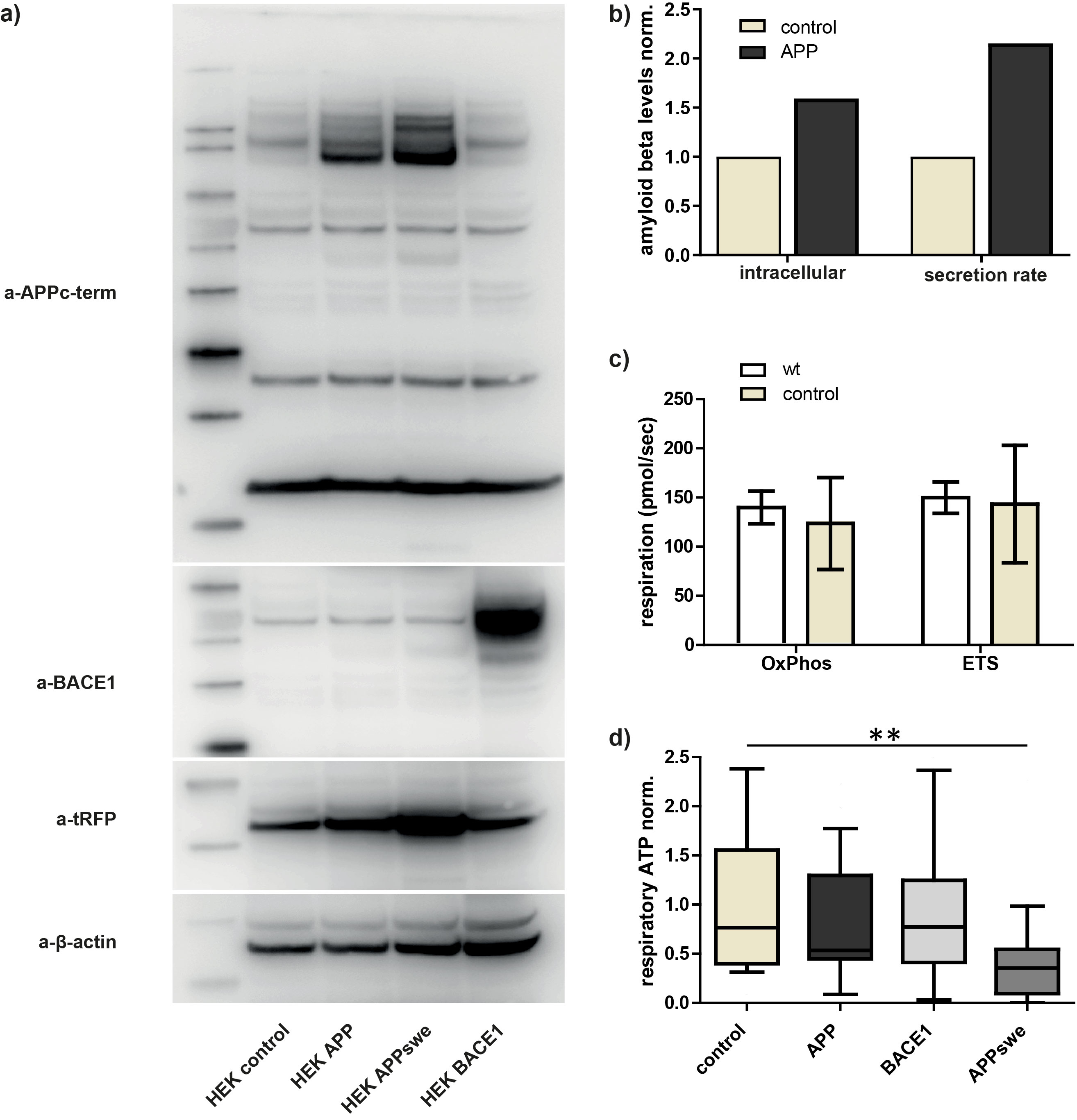

Supplement: Supplementary file 2 [file Image1.JPEG]

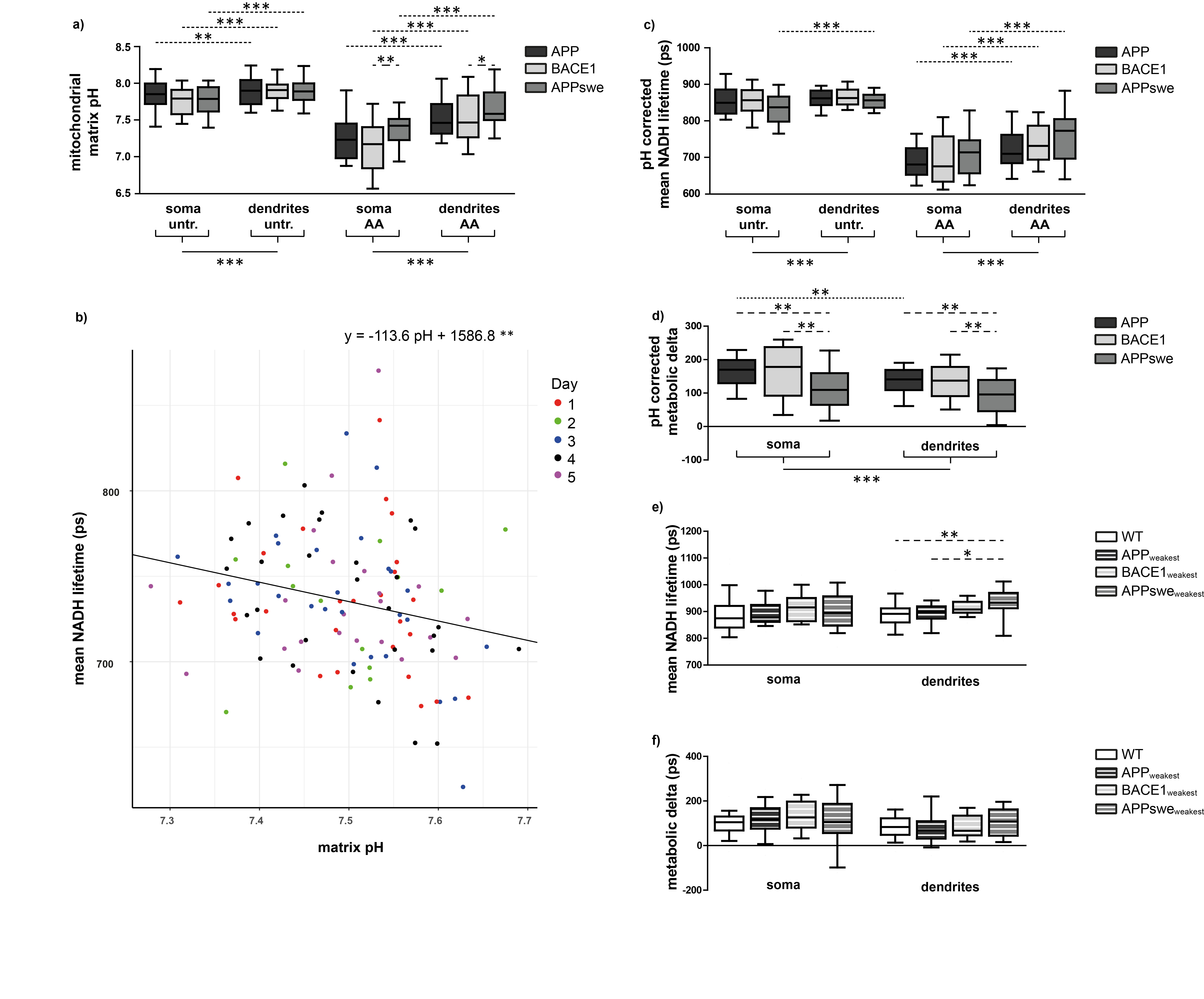

Supplement: Supplementary file 3 [file Image2.JPEG]
